# Supporting high-quality enrollment decision-making in clinical research: development of a novel informed consent communication framework and curriculum

**DOI:** 10.1186/s12874-026-02885-2

**Published:** 2026-07-25

**Authors:** Stephanie A. Kraft, Kathryn M. Porter, Abigail Brickler, Sylvia Mazuera, Holly A. Taylor, Luke Gelinas, Devan M. Duenas, Jennifer C. Tsing, Cynthia George, Tamar Krishnamurti, Aisha T. Langford, Rebecca Mueller, Kathleen J. Ramos, Holly H. Võ, Elliott M. Weiss, Benjamin S. Wilfond

**Affiliations:** 1 Department of Bioethics and Decision Sciences, Geisinger College of Health Sciences, 100 N. Academy Ave, Danville, PA 17822 USA; 2 Treuman Katz Center for Pediatric Bioethics and Palliative Care, Seattle Children’s Research Institute, Seattle, WA USA; 3 Community member, Carrollton, TX USA; 4 Department of Bioethics, Clinical Center National Institutes of Health, Bethesda, MD USA; 5 Advarra IRB, Columbia, MD USA; 6 Cystic Fibrosis Foundation, Bethesda, MD USA; 7 Division of General Internal Medicine, University of Pittsburgh, Pittsburgh, PA USA; 8 Department of Family Medicine and Public Health Sciences, Wayne State University, Detroit, MI USA; 9 Division of Translational Medicine and Human Genetics, Department of Medicine, University of Pennsylvania, Philadelphia, PA USA; 10 Division of Pulmonary, Critical Care, and Sleep Medicine, Department of Medicine, University of Washington, Seattle, WA USA; 11 Department of Pediatrics, University of Washington, Seattle, WA USA

**Keywords:** Informed consent, Recruitment, Clinical trials, Communication, Research relationships

## Abstract

**Background:**

In clinical trials, the process of informed consent is intended to support prospective participants in making enrollment decisions that are well informed and aligned with their values and preferences. Despite the importance of relationship building during the consent process, there is little structured guidance for researchers on how to effectively facilitate informed consent conversations with prospective participants. This project aimed to develop and implement a relationship-based communication framework for clinical researchers, with the ultimate goal of supporting prospective participants in making values-aligned decisions.

**Methods:**

The ENgaging with Values to Inform research enrollment deciSIONs (ENVISION) study was launched to improve the process of research enrollment decision-making within the context of a multisite cystic fibrosis clinical trial. The ENVISION team sought input from individuals with cystic fibrosis, researchers, institutional review board leaders, and clinical research leaders to understand key issues in consent communication in the context of cystic fibrosis trials. A working group of the ENVISION team incorporated these viewpoints into the development of a consent communication framework, which was guided by consideration of elements of the Ottawa Decision Support Framework and incorporated established communication strategies. The communication framework and an accompanying training curriculum were finalized through iterative review with the working group and the full team.

**Results:**

The development process resulted in a four-component, values-oriented communication framework, the RIVER framework: Relationship (forming a connection with the prospective participant), Information (sharing about the study), Values Exploration (eliciting and examining relevant values), and Resolution (confirming a values-aligned decision). Each component includes considerations to support prospective participants in making values-aligned decisions. RIVER is implemented through a training curriculum designed for research coordinators. The training curriculum consists of two 2-hour sessions that include didactic teaching, group discussion, and role-play scenarios, which provide an opportunity to practice real-world clinical research enrollment conversations.

**Conclusion:**

The RIVER framework offers a structured approach to communication during clinical research consent conversations that is designed to enhance voluntary, informed decisions. Ongoing evaluation of the framework and training curriculum will inform opportunities for future revisions, adaptations, and broader dissemination.

**Supplementary Information:**

The online version contains supplementary material available at https://doi.org/10.1186/s12874-026-02885-2.

## Background

The process of informed consent is a critical ethical benchmark for clinical research [1]. An effective informed consent discussion supports informed decision-making among prospective participants [2] and advances ethical goals such as autonomy, values alignment, and trust [3]. In theory, a well-designed informed consent process allows a prospective research participant to learn about a study, consider whether they want to take on the study’s balance of risks, benefits, and burdens, and make a decision about participation that aligns with their personal values, preferences, and constraints. In practice, however, informed consent processes often fail to support participant understanding of key study information. Consent forms can be overly complex, which can lead to information overload, undermine comprehension [4–6], and ultimately compromise informed decision-making. The addition of a regulatory requirement to highlight a study’s “key information” at the start of the consent form was intended to help address these issues, but there is evidence that key information sections are falling short of their goals [7].

While continued efforts to improve clarity in consent forms are important, enhancing the quality of the accompanying conversations is perhaps even more critical. High-quality conversations have been shown to support participant understanding of study information [8, 9] and play a major role in enrollment decision-making [10, 11]. These conversations provide a forum in which prospective participants can identify and consider their values, priorities, and constraints as they relate to potential study enrollment. Prior studies suggest that some prospective participants make enrollment decisions early in the consent process, based more on interactions with research team members than their review of the consent form [11, 12]. However, research team members who facilitate these conversations often lack guidance on how to structure consent conversations that effectively draw out prospective participants’ preferences. In the absence of guidance, these conversations typically focus primarily on information disclosure, resulting in missed opportunities to explore the extent to which an individual’s values align—or do not—with study participation [13]. This project sought to address this gap by providing a structured approach to values-oriented consent conversations such that each prospective participant’s enrollment decision reflects their informed preferences. 

Techniques from clinical communication and shared decision-making practices may be helpful to support prospective participants in making values-aligned decisions about research participation [14]. For example, one study identified the use of shared decision-making during the enrollment process as a factor contributing to improved patient satisfaction with clinical trial decision-making [15]. Research team members have highlighted the importance of establishing and maintaining trusting relationships with prospective participants as part of the consent approach [16], as well as the value of tools like teach-back methods to improve understanding [17]. Similarly, participants across different research settings have emphasized the importance of experiencing respect and developing trusting relationships through interpersonal interactions when approached to consider enrollment in research [18, 20]. Respectful research relationships may be especially important when engaging with prospective participants amidst widespread skepticism of science and medicine [21, 22]. Additionally, respectful interactions have value on a population level insofar as they may enhance overall rates of enrollment, retention, and adherence to study protocols [23].

Relationship-based communication training with clinical teams has shown potential to enhance patient care [24]. Systematic training in communication that is attuned to relationship building, however, is not common in the clinical trials setting. Some prior work has begun to address the need for relationship-based research communication training for individuals who will be approaching prospective participants [25, 26]. Still, there are further opportunities to enhance communication training, for example, by developing approaches to encourage prospective participants to explore the reasons why they may or may not want to participate [27]. This project aimed to incorporate lessons learned from clinical shared decision-making and relationship-based communication into clinical research communication. Building on prior work in clinical [24] and research [25, 26] settings, this project created a novel, relationship-based communication framework for research team members approaching prospective clinical trial participants. This manuscript presents an overview of the communication framework development process, beginning with the overarching project goals and structure, summary of preliminary engagement with key constituents, and structured identification of opportunities to enhance consent conversations. It then describes the resulting framework as well as a training curriculum to facilitate and encourage uptake. The manuscript concludes with reflections on opportunities for further revisions and evaluation.

## Methods

### The ENVISION project

Development of the novel communication framework was a component of the ENgaging with Values to Inform research enrollment deciSIONs (ENVISION) study. The overarching goal of ENVISION is to develop and implement tools to improve the process of research enrollment decision-making such that prospective participants can make informed decisions that align with their values and preferences. The ENVISION approach includes the communication framework and training curriculum described here, as well as a streamlined digital informed consent tool that will be described elsewhere.

The ENVISION team includes a core research team of investigators and staff with expertise in research ethics and clinical trials, as well as an external steering committee comprised of experts in topics including informed consent, clinical trials, research coordination, decision science, implementation science, regulatory science, and lived experience of cystic fibrosis. Several working groups were formed to lead distinct components of the overall project and report back to the broader team. Working group members included core team and steering committee members with relevant expertise. Additional scholars were recruited to join the working groups as needed.

The ENVISION project is currently piloting its approach as an embedded study within the STOP360 (Standardizing Treatments for Pulmonary Exacerbations) trial, a multisite clinical trial comparing the effectiveness of two antibiotic regimens in treating pulmonary exacerbations in people with cystic fibrosis [28]. ENVISION is partnering with 12 U.S. STOP360 sites to implement the ENVISION approach and train research coordinators in the communication framework. Planned outcomes for this pilot study will assess feasibility, acceptability, and appropriateness of the communication training, fidelity of actual research approaches to the communication framework, and qualitative experiences of prospective participants who were approached by an ENVISION-trained coordinator. The findings of the pilot will be shared in a future manuscript.

### Understanding key constituent priorities

As part of the development process for the communication framework, the ENVISION team sought feedback from constituent groups with relevant views on the consent process for the STOP360 study. These groups included people with cystic fibrosis (*n* = 11 in 4 groups), STOP360 study investigators and staff (*n* = 14 in 3 groups), Institutional Review Board (IRB) leaders from 3 organizations (*n* = 10 in 3 groups), and clinical research leaders from the Cystic Fibrosis Foundation Therapeutics Development Network and Data Safety Monitoring Board (*n* = 5 in 1 group). Discussions were designed to provide rapid, iterative feedback on the development of the ENVISION approach. Groups responded to specific questions about communication during the consent process that included which topics, beyond the information provided about the study, should be explored during the consent conversation and whether and how to support prospective participants in making a decision. Discussion groups took place between August and October 2024. One or more notetakers were present during each discussion group to document key points; notes were subsequently collated to facilitate integration into revisions of the ENVISION approach and are summarized below.

Key areas of feedback on consent communication emphasized two broad areas: the value of exploring prospective participants’ individualized needs during consent conversations, and the importance of ensuring participants feel empowered to make their own decisions without pressure from the research team. All constituent groups agreed that a robust and transparent conversation facilitates a deep understanding of the study and its risks, benefits, and burdens, as well as the extent to which participation might align with an individual’s values and preferences. Suggested topics to explore included prospective participants’ life priorities beyond medical topics, their important relationships, their prior familiarity with research and understanding of research goals, and how the study treatments may compare to their prior experiences.

To achieve communication goals, researchers and IRB leaders highlighted the value of training team members who would be engaging prospective participants in consent discussions in specific skills such as active listening and being attuned to body language. Individuals with cystic fibrosis and IRB leaders both expressed that research coordinators approaching prospective participants about study enrollment could facilitate decision-making by noting and reflecting on values shared by those prospective participants during the conversation. The groups did not think that framing a direct recommendation about enrollment based on those stated values would be appropriate. Rather, there was emphasis on making clear that prospective participants are solely responsible for deciding whether to participate. Researchers similarly emphasized the importance of making sure the prospective participant feels that enrollment is a choice, given the power imbalance inherent to consent conversations and the pressure to enroll that can sometimes be felt. This collective feedback was incorporated into the communication framework, as described below.

### Identifying gaps in consent communication

The ENVISION working group on communication framework and training development was led by one of the two ENVISION principal investigators (SAK) and met monthly over a period of approximately 18 months. The group was tasked with developing a communication framework to guide the informed consent conversation, creating materials and teaching tools to facilitate use of the framework, and implementing the framework and training in the context of the STOP360 study. Research coordinators were selected as the target audience for the framework and training, as individuals in this role are typically tasked with conducting consent conversations. The working group used a structured approach to create an initial communication framework guided by the Ottawa Decision Support Framework, which is commonly used to guide development of clinical decision aids, particularly in settings where decisions depend on the values of those deciding [29]. The group used the structure of the Ottawa Decision Support Framework to outline relevant considerations in prospective participants’ decision-making process and, from these, identify important components of communication during this process. Feedback from key constituents was incorporated into the outline of the communication process. In addition, the group drew on their respective areas of expertise and prior literature review to identify prospective research participants’ decisional needs (e.g., personal connection [18, 19], communication that facilitates information processing [30, 31], identification of concerns and preferences [32]), key outcomes for high-quality decision-making (e.g., trust [33], understanding [34], partnership [35]), and potential interventional supports to address needs and work toward targeted outcomes (e.g., research relationship building [26], values exploration via open communication [24]), incorporating established patient-centered communication strategies [36, 37], where relevant. The group then organized identified needs, outcomes, and supports into categories and, through an iterative review process across multiple meetings, formed a novel four-part framework for relationship-based communication to support values-aligned enrollment decision-making. The draft framework was iteratively refined with periodic feedback from the ENVISION steering committee and core research team.

## Results

### RIVER framework

**Fig. 1:**
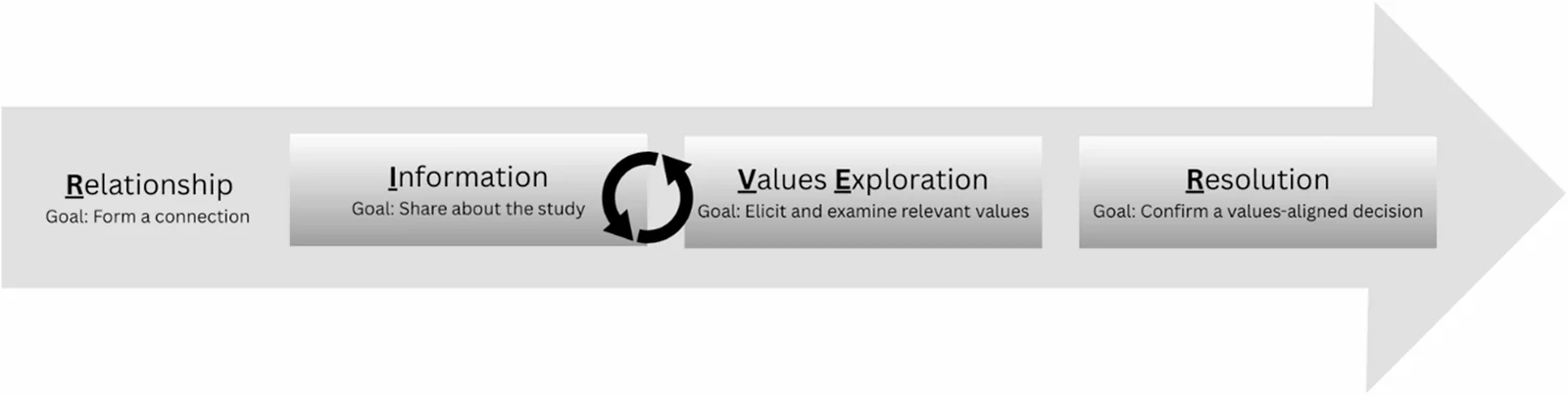
RIVER framework for values-oriented consent communication

The development process produced a values-oriented communication framework for clinical research enrollment conversations called the RIVER framework, which includes four components: Relationship, Information, Values Exploration, and Resolution (Fig. 1).

The four components of RIVER generally flow in order but may be modified based on a prospective participant’s individual needs, and they may be revisited throughout one or more conversations. For example, opportunities for continued relationship building may arise throughout a discussion, and information and values exploration may be interwoven as the prospective participant considers their values alongside the study information. RIVER is designed for consent conversations about initial enrollment but may also be used to support decision-making in response to new information that warrants re-consent during an ongoing study. The four components are described below and example language shown in Table 1.

**Table 1:** RIVER components and example language

Component	Details	Example language
Relationship	Introduce yourself to everyone in the room. Avoid making assumptions about people’s relationships.	• *Hi*,* I’m [name and role]. I’m here to talk with you about a potential research study.* • *Are you [name]? It’s nice to meet you. How would you like me to address you?* • *Who have you brought with you?*
	Acknowledge the prospective participant’s personal and clinical situation and share that you are there to support them.	• *I understand from your doctor that [clinical status]. How are you feeling about this? What previous experiences have you had with this?* • *Your doctor*,* [name]*,* shared that there is a research study that you might be interested in. I’m here to talk with you about the study and support you in thinking about whether it might be a good fit for you.*
	Check if it’s a good time to talk about research. If it’s not, set a time to follow up.	• *Would you be interested in talking with me about this research study?* • *This discussion usually takes about [estimated time]. Is this a good time?* • *What other things do you want to attend to before we talk?*
Information	Review the informed consent in small sections. Explore topics the prospective participant is most interested in and check in on their understanding.	• *I’m curious to hear what stood out to you about the information we’ve reviewed so far.* • *To help me be sure I’m explaining everything as clearly as I intend*,* would you mind sharing in your own words what we’ve covered so far?*
	Elicit and list the prospective participant’s questions. Spend more time on topics where they have questions and check in after key parts.	• *I want to take some time to check what questions you have. People have questions about lots of different things in the study*,* such as how much time it takes or the risks involved.* • *Let’s make a list of your questions so we can make sure we address all of them.*
Values Exploration	As you review the study information, explore what reasons they are thinking about for joining or not.	• *We discussed a few examples of reasons why people might want to join or not join the study. Which reasons resonate with you?* • *It sounds like [reflect reasons] are important to you. Do these make you lean in one direction or the other at this point?*
	Elicit priorities that might be relevant to the study. These often include health-related priorities and other life priorities during the study period.	• *I’m interested in hearing what you’re thinking and how this study might/might not fit into your life right now. When you think about whether this study is something you want to take part in*,* what things are important to you?* • *Thanks for sharing [reflect]. If you imagined joining the study*,* how would that fit with what you’ve shared with me?*
	Elicit any concerns or barriers to participation and discuss any ways these could be navigated.	• *How would the schedule of this study work for you? What could we do to help with this?* • *What is your support system for participating in this study? How will you get to visits? What could we do to help with this?* • *Is there anything else you’ve heard so far that might be an issue for you?*
Resolution	Reflect, confirm, and validate the person’s key reasons, priorities, and concerns/barriers (and ways to address them). Check in on their decision.	• *We’ve talked about [reflect]. Did I get that right? Do you want to talk more about anything?* • *How are you feeling about the study?*
	If they: • *Say yes*: Thank them for the conversation and provide them next steps for the study. • *Say no*: Thank them for their time/consideration. • *Express conflict*: Validate their uncertainty and further explore their values. • *Need time*: Check in on any next steps they may want to take before deciding.	

#### Relationship

The research coordinator begins (or, if a relationship has previously been established, continues) to build a respectful relationship with the prospective participant, with the goal of building rapport and fostering a trusting connection that will facilitate ongoing discussion about the study. Specific steps include introducing themselves to the prospective participant and any family or friends they have with them; acknowledging the prospective participant’s personal and clinical situation and that they are there to support them; and checking to see if it is a good time to talk and, if not, setting a time to follow up.

#### Information

The research coordinator reviews the study information with the prospective participant, drawing on any written consent materials and following a structured process that incorporates active listening skills and evidence-based communication practices like teach-back [38, 39]. The research coordinator begins by reviewing the “key information” section of the informed consent form, after which they check in about the prospective participant’s understanding of the study (e.g., whether they adequately grasp the study activities, main risks and benefits and respective likelihoods, and distinction from clinical goals), elicit and list any questions they might have, and explore what they may be thinking about enrollment, drawing on examples of reasons why someone might or might not want to enroll. At this stage in the conversation, some may be ready to decline participation based on the overview of the study as described in the key information. For those who are interested in learning more, the research coordinator continues to review the study details with the prospective participant. Through this conversation, the research coordinator shares information in small sections to facilitate back-and-forth discussion, focusing extra attention on the topics about which the prospective participant has questions and/or otherwise indicates are most important to them.

#### Values exploration

The research coordinator elicits and explores the prospective participant’s values that relate to whether they might want to enroll, which may include their general attitudes about risk, uncertainty, and the value of research participation. This component focuses on reasons an individual may be considering for joining or not joining the study by explicitly integrating information about the individual’s priorities and concerns into the context of the study. This component also includes eliciting the prospective participant’s health-related and other life priorities that might be relevant to the study, for example professional or family commitments during the study period. The research coordinator also elicits any concerns or barriers to participation and discusses the extent to which there may be mitigating factors that could help address these. Through this process of values exploration, the research coordinator’s goal is to support the prospective participant in thinking through whether their values, priorities, and concerns align with study enrollment.

#### Resolution

The research coordinator shares their interpretation of the participant’s key reasons, priorities, and concerns or barriers previously identified through conversation. After confirming and validating these key discussion points, the research coordinator checks if the prospective participant is ready to make a decision. If the prospective participant needs more time, the research coordinator validates any conflict or uncertainty, checks in on next steps, and proposes a follow-up plan.

### The RIVER training curriculum

After developing the framework, the working group considered how best to train professional research coordinators in implementing the communication framework. The working group identified potential training approaches that would address identified gaps in current approaches to consent communication, drawing on theories for professional learning including adult learning theory [40] and experiential learning [41], as shown in Table 2. These theories were selected as optimal for training research coordinators who may have a wide range of professional experiences with consent conversations as well as time constraints in their professional roles.

**Table 2:** Training elements and their theoretical bases

Training element	Theoretical basis	Application of theory to training element
Pre-session materials	• Self-concept (adult learning theory)	Materials allow for independent, self-driven review (self-concept).
Didactics	• Need to know/why (adult learning theory) • Intrinsic motivation (adult learning theory)	Didactics describe justification for approach (need to know/why) and anticipated impact on interactions with prospective participants (intrinsic motivation).
Group discussions about past experiences and anticipated implementation challenges	• Role of experience (adult learning theory) • Abstract conceptualization (experiential learning)	Discussions build on existing expertise (role of experience) and ask learners to learn from past experiences (abstract conceptualization).
Role plays	• Readiness to learn (adult learning theory) • Orientation to learning (adult learning theory) • Concrete experience (experiential learning)	Role plays are oriented to professional situations (readiness to learn), use a problem-based approach (orientation to learning), and provide a realistic encounter in which to implement skills (concrete experience).
Peer feedback and group reflections on role plays	• Reflective observation (experiential learning)	Learners reflect on an actual experience (reflective observation).
Ongoing opportunities for reflection during implementation	• Active experimentation (experiential learning)	Learners try out what they have learned in practice (active experimentation).

**Fig. 2:**
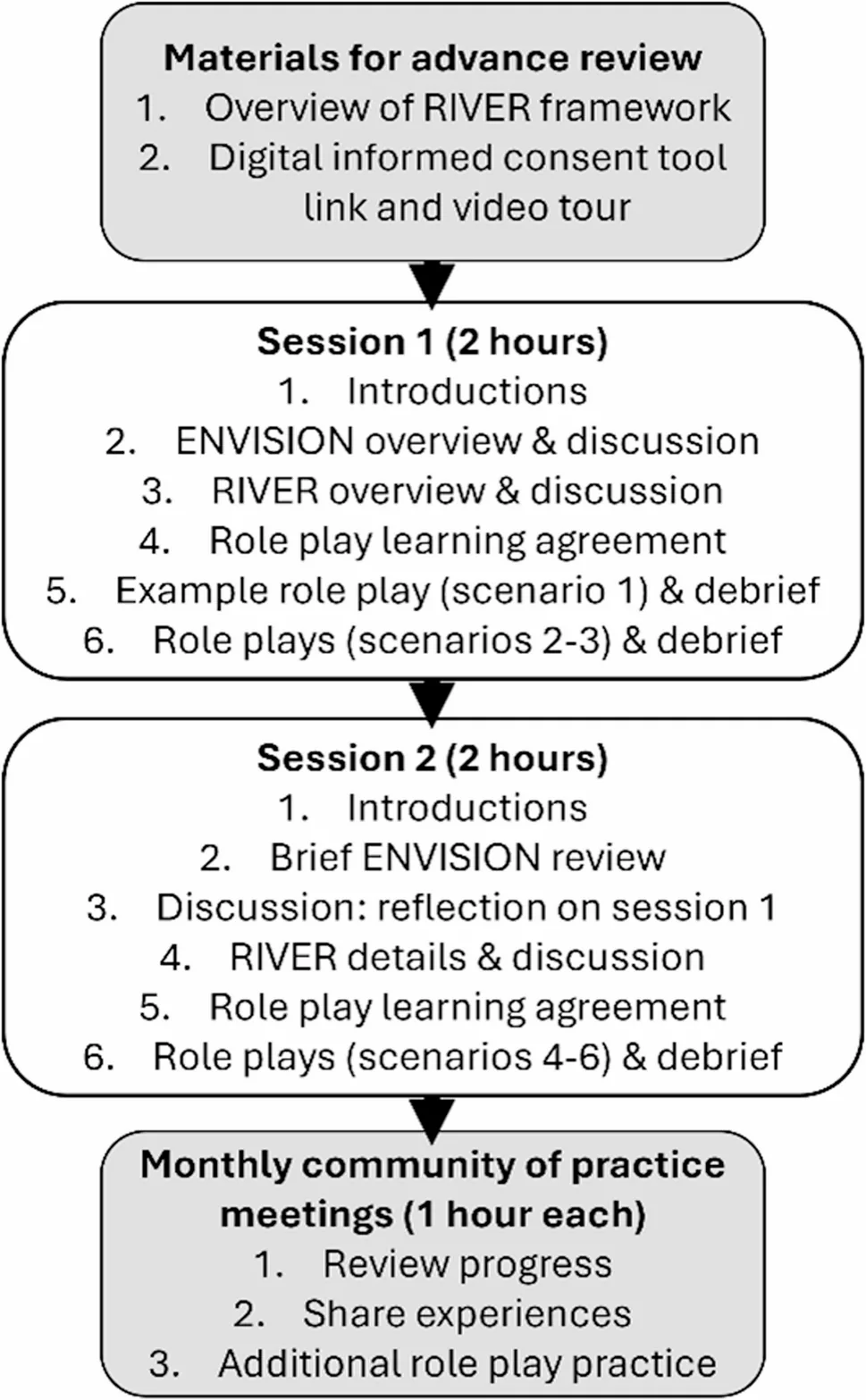
RIVER training curriculum

The training curriculum (Fig. 2) was structured to include two 2-hour sessions, each with a combination of didactic teaching, group discussions, and role-play opportunities. Each role-play scenario was designed to raise a distinct set of clinical considerations, research-related values, and conversational goals, as shown in Table 3. Prior to the role-play exercises, a simulation learning agreement was presented to encourage engagement in the simulation activities, respectful and constructive critiques, and confidentiality. 

Role plays were hypothetical but informed by knowledge of patients with cystic fibrosis that include information related to three components: personal (e.g., interests and activities, family, work), clinical status (e.g., medication use, pulmonary function, recent exacerbation and antibiotic history), and research experience and attitudes. The descriptions varied in complexity and were designed to reflect a range of real-world scenarios to support practice conversations using RIVER. The role-play exercises in the first training session emphasized relationship building and asked research coordinators to initiate a consent conversation and begin the process of information sharing and values exploration. A pre-recorded role-play conversation from the ENVISION team was shared with trainees prior to the first role-play exercise as an example of how to use the RIVER framework in practice. Role-play exercises in the second training session emphasized coming to a resolution that built on the information and values discussed throughout the conversation.

**Table 3:** Role play components

Scenario	Personal	Clinical status*	Research experience and attitudes
Session 1: emphasis on relationship building			
1 (example)	Alex, age 30, single, works at a tech company, has a dog and likes rock climbing	On modulator, mild lung disease, averages 3 mild pulmonary exacerbations/year, treated with oral antibiotics, no recent hospitalizations	Very open to talking about research and responsive to questions
2	Sam, age 40, married with 7-year-old child, stay-at-home parent, active in church and PTA	Not on modulator due to side effects, moderate lung disease requiring inhaled antibiotic every other month, averages 3 pulmonary exacerbations/year and 1–2 hospitalizations for IV antibiotics	Has not previously been engaged/approached about research, hesitant but open when asked the right questions
3	Quinn, age 40, married with 16-year-old twins, works retail part-time, rides an e-bike	On modulator, moderate lung disease, averages 1 pulmonary exacerbation/year, treated with oral antibiotics, no recent hospitalizations	Has lots of questions, stressed about potential fit with life, wants to involve partner and can’t decide right away
Session 2: emphasis on coming to a resolution			
4	Jordan, age 20, single, works for outdoor equipment design company, travels internationally, lives with parents and younger siblings	On modulator, normal lung function with minimal lung disease, has cystic fibrosis-related diabetes, hasn’t needed any antibiotics in the last year	Doesn’t know much about research, doesn’t know what questions to ask; learned about study and reviewed consent with principal investigator earlier today but didn’t say much
5	Cameron, age 25, attends community college and works as a nanny, recently moved into an apartment with a roommate, enjoys online gaming	Not eligible for modulators, moderate lung disease, 3 pulmonary exacerbations requiring hospitalization for IV antibiotics in the last year, recently started medication for anxiety	Prior research relationship with research coordinator of 2 years; reviewed most of the consent with research coordinator earlier today and was curious about the study, expressing a desire to give back, but also hesitant about bad things happening in research; asked for time to think and look over information
6	Terry, age 62, recently retired as an engineer, married with no children, likes woodworking and contemporary music	Refused modulator, mild lung disease, has chronic sinus disease which has required surgery, hasn’t had a pulmonary exacerbation in 2 years	Met with research coordinator and discussed key information earlier today; was confrontational and got upset about the implication their doctor doesn’t know what’s best but also shared a view that doctors should use fewer antibiotics; asked to talk to doctor first

*Role play scenarios assume that research coordinators have basic knowledge about cystic fibrosis. Cystic fibrosis is a genetic disease that affects a chloride and bicarbonate channel in many cells of the body, impacting the exchange of sodium and water with accumulation of viscous mucus in multiple organ systems. The primary impact is seen in the lungs where chronic infection and inflammation lead to a decrease in lung function ranging from mild to severe. Infections can periodically worsen, exacerbating symptoms and requiring treatment with antibiotics and sometimes hospitalization. In the past two decades, research has resulted in the approval of a class of drugs known as modulators, which modify the dysfunctional cystic fibrosis protein at the cellular level, resulting in improvement in the chloride channel function. The most recently approved modulators significantly decrease the amount of mucus in the lungs, thereby reducing infection/exacerbations and improving lung function

Before the first training session, research coordinators received written materials to familiarize themselves with the RIVER framework (see RIVER Brochure, Supplemental File). After training, coordinators were invited to attend monthly virtual community of practice meetings for ongoing reflection and practice. The concept of a community of practice is intended to foster a learning community in which professionals build social connections to support the exchange of knowledge and experiences [42].

## Discussion

The RIVER framework reflects a structured approach to promoting relationship-based communication in clinical research recruitment. In doing so, it aims to enhance research teams’ ability to engage with prospective participants during informed consent conversations and support prospective participants in making voluntary decisions that align with their individual values and preferences. Lessons learned from RIVER’s ongoing implementation will inform future refinement to ensure it is meeting its goals.

By building a foundation of a trusting relationship and emphasizing values exploration throughout the process of information sharing, RIVER aims to support prospective participants’ understanding of required elements of consent, thorough consideration of personally relevant factors, and ability to make enrollment decisions that are aligned with their values. Despite these goals, some researchers or regulators may feel discomfort at focusing on relationship building, as it may seem counter to traditional views that research conversations should be focused primarily on information provision. Some may worry that relationship building could lead to undue influence on participant decisions in certain circumstances [43, 44]. In a prior interview study, research coordinators discussed the importance of addressing power dynamics during the process of building relationships with prospective participants, describing strategies to reduce pressure that included emphasizing the prospective participant’s right to make a voluntary decision, providing sufficient time, and clarifying the distinction between clinical and research decisions [16]. Future expansions of the RIVER framework and training could more explicitly incorporate strategies like these to address power imbalances, particularly when implemented with populations who may have heightened vulnerability to undue influence. Discussion group feedback that contributed to the development of the RIVER framework highlighted the importance of both ensuring individuals feel free to make their own choice and supporting exploration of a broad range of values that may inform one’s preferences. These components reflect both negative and positive obligations to promote prospective participants’ autonomy [45]; that is, researchers should take steps to both avoid interfering with autonomous decisions and simultaneously offer the tools, information, and support needed to enhance autonomous decision-making. While the RIVER framework’s emphasis on relationship building and values exploration is intended not only to avoid undue influence but to enhance voluntariness by creating a foundation for open dialogue, teams who implement RIVER should monitor for the possibility that prospective participants experience pressure and, if identified, revise any problematic components of the framework or its implementation. Future work may consider, for example, guidance on how researchers can identify and respond to indications of pressure, including pausing the conversation if needed and returning at another time.

RIVER was developed in the context of a clinical trial for individuals with cystic fibrosis, but it offers a general framework that can be adapted for other study contexts. The decisional needs and outcomes identified through the RIVER development process are not unique to cystic fibrosis clinical trials; however, different trial settings may warrant tailoring prior to implementation. Input from those with lived experience of the condition(s) under study will be key for such adaptation, as patients with different conditions may need different amounts and types of information. For example, a patient population without as much prior clinical engagement may require additional discussion of how research participation is different from receiving standard care, in contrast to those with medical conditions where research is routinely integrated with clinical care. Expanding on informational strategies to mitigate the possibility that participants overestimate the likelihood of personal clinical benefit from enrolling [46] may be particularly important in such cases. Modifications may also be warranted in settings where research activities are integrated within clinical care. For example, an integrated approach that builds research informed consent into usual clinical practice may streamline the consent process and facilitate collection of real-world data in pragmatic clinical trials [47]; such an approach could be readily adapted to incorporate the key elements of RIVER and training could be updated to include language specific to the integrated setting. Additionally, RIVER was developed with the expectation that research coordinators would be leading the majority of the informed consent conversations and thus would be the primary audience for the framework and training, reflecting the lack of consistently available training across the research coordinator profession [16]. However, there is variation between trials, as well as between sites in the same trial, as to which member or members of the team—including members of the prospective participant’s clinical care team—introduce the study and discuss different components. RIVER and the associated training may need to be adjusted for investigators or other research team members with different professional backgrounds and roles.

Outcomes of RIVER’s ongoing piloting, including lessons captured through implementation, will inform future modification of the framework and recommendations for broader dissemination across other clinical trials. Feedback regarding the feasibility of the time demands will be especially important to consider. A more deliberative, relationship-focused conversation may increase the time that informed consent conversations take. Some prospective participants may find this increased time valuable, while others may find it burdensome; similarly, research team members may need to balance other obligations. Additionally, the training requires research coordinators to attend 4 hours of live virtual programming plus monthly meetings for the duration of the study; the balance of benefits and burdens of each training component should also be considered. Future trainings may be able to implement certain components asynchronously, e.g., didactics via pre-recorded videos and streamlined live role-play practices. Assessments should evaluate which components of the training are most effective so future training can optimize both efficacy and feasibility. 

Finally, the RIVER framework was developed only in English. While bilingual research coordinators using RIVER can adapt the framework for conversations in other languages, there may also be opportunities for improved translation earlier in the process. For example, based on common languages spoken by potential participants, question prompts could be prepared and reviewed in advance. While consent conversations using the RIVER framework are intended to support written consent materials, the quality of the conversation may be particularly important for populations that use a language other than English [48]. Incorporating additional cultural considerations into RIVER may also be warranted, especially for studies that plan to recruit individuals or populations with specific cultural norms around communication and relationship-building. Differential experiences across language and sociocultural subgroups will be important to monitor in the implementation of RIVER and may reveal opportunities for future improvement.

## Conclusions

The RIVER framework is a structured, relationship-based communication framework designed to support those approaching prospective research participants to facilitate values-aligned decisions about clinical trial enrollment. Evaluation of implementation with research teams and impact on prospective participants’ decision-making processes will be a critical next step when considering broader dissemination of this framework.

## Supplementary Information

Supplementary Material 1.

## Data Availability

No datasets were generated or analysed during the current study.

## Abbreviations

ENVISION: Engaging with Values to Inform research enrollment deciSIONs
RIVER: Relationship, Information, Values Exploration, Resolution
STOP360: Standardizing Treatments for Pulmonary Exacerbations
IRB: Institutional Review Board

## References

[1] Emanuel EJ, Wendler D, Grady C What makes clinical research ethical? Jama. May 24–31. 2000;283(20):2701-11. 10.1001/jama.283.20.2701. 10819955

[2] National Commission for the Protection of Human Subjects of Biomedical and Behavioral Research. The Belmont report: Ethical principles and guidelines for the protection of human subjects of research. 1979. https://www.hhs.gov/ohrp/regulations-and-policy/belmont-report/read-the-belmont-report/index.html. 25951677

[3] Dickert N, Eyal N, Goldkind S, et al. Reframing Consent for Clinical Research: A Function-Based Approach. Am J Bioethics: AJOB. 2017;17(12):3–11. 10.1080/15265161.2017.1388448. 29148951

[4] Kass NE, Chaisson L, Taylor HA, Lohse J. Length and Complexity of US and International HIV Consent Forms from Federal HIV Network Trials. J Gen Intern Med. 2011;26(11):1324–8. 10.1007/s11606-011-1778-6. PMC3208469 21748435

[5] Tam NT, Huy NT, le Thoa TB, et al. Participants’ understanding of informed consent in clinical trials over three decades: systematic review and meta-analysis. Bull World Health Organ. 2015;1(3):186–h98. 10.2471/blt.14.141390. PMC4371493 25883410

[6] Emanuel EJ, Boyle CW. Assessment of Length and Readability of Informed Consent Documents for COVID-19 Vaccine Trials. JAMA Netw Open. 2021;1(4):e2110843. 10.1001/jamanetworkopen.2021.10843. PMC8082317 33909052

[7] Gelinas L, Morrell W, Tse T, Glazier A, Zarin DA, Bierer BE. Characterization of key information sections in informed consent forms posted on ClinicalTrials.gov. J Clin Transl Sci. 2023;7(1):e185. 10.1017/cts.2023.605. PMC10514692 37745937

[8] Nishimura A, Carey J, Erwin PJ, Tilburt JC, Murad MH, McCormick JB. Improving understanding in the research informed consent process: a systematic review of 54 interventions tested in randomized control trials. BMC Med Ethics 2013;23:14:28. 10.1186/1472-6939-14-28. PMC3733934 23879694

[9] Tamariz L, Palacio A, Robert M, Marcus EN. Improving the informed consent process for research subjects with low literacy: a systematic review. J Gen Intern Med. 2013;28(1):121–6. 10.1007/s11606-012-2133-2. PMC3539038 22782275

[10] Ulrich CM, Ratcliffe SJ, Hochheimer CJ, et al. Informed Consent among Clinical Trial Participants with Different Cancer Diagnoses. AJOB Empir Bioeth. 2024;15(3):165–77. 10.1080/23294515.2023.2262992. PMC11492203 37921867

[11] Kraft SA, Porter KM, Duenas DM, et al. Assessing Parent Decisions About Child Participation in a Behavioral Health Intervention Study and Utility of Informed Consent Forms. JAMA Netw Open. 2020;1(7):e209296. 10.1001/jamanetworkopen.2020.9296. PMC7395235 32735337

[12] Weiss EM, Guttmann KF, Olszewski AE, et al. Parental Enrollment Decision-Making for a Neonatal Clinical Trial. J Pediatr. 2021;239:143–e1493. 10.1016/j.jpeds.2021.08.014. PMC8610170 34400207

[13] Miller VA, Baker JN, Leek AC, Drotar D, Kodish E. Patient involvement in informed consent for pediatric phase I cancer research. J Pediatr Hematol Oncol. 2014;36(8):635–40. 10.1097/MPH.0000000000000112. PMC4116468 24487916

[14] Weiss EM, Wilfond BS, Kraft SA. Towards better enrollment decision-making for perinatal clinical research: Reconsidering recruitment and consent processes to support family values and preferences. Semin Perinatol. 2025;49(3):152055. 10.1016/j.semperi.2025.152055. PMC12407186 40404236

[15] Chang WW, Wu MT, Chang YC, Hu WY. The mediating effect of shared decision-making in enhancing patient satisfaction with participation in cancer clinical trials. Asia Pac J Oncol Nurs. 2023;10(8):100265. 10.1016/j.apjon.2023.100265. PMC10372169 37519403

[16] Kraft SA, Porter KM, Sullivan TR, et al. Relationship building in pediatric research recruitment: Insights from qualitative interviews with research staff. J Clin Transl Sci. 2022;6(1):e138. 10.1017/cts.2022.469. PMC9794958 36590359

[17] O’Sullivan L, Feeney L, Crowley RK, Sukumar P, McAuliffe E, Doran P. An evaluation of the process of informed consent: views from research participants and staff. Trials Aug. 2021;18(1):544. 10.1186/s13063-021-05493-1. PMC8371296 34407858

[18] Kraft SA, Rothwell E, Shah SK, et al Demonstrating ‘respect for persons’ in clinical research: findings from qualitative interviews with diverse genomics research participants. J Med Ethics. 2020;6. 10.1136/medethics-2020-106440. PMC8021602 33023975

[19] Kraft SA, Duenas DM, Shah SK. Patient priorities for fulfilling the principle of respect in research: findings from a modified Delphi study. BMC Med Ethics. 2023;21(1):73. 10.1186/s12910-023-00954-5. PMC10512546 37735658

[20] Kelley M, James C, Alessi Kraft S, et al. Patient Perspectives on the Learning Health System: The Importance of Trust and Shared Decision Making. Am J Bioeth. 2015;15(9):4–17. 10.1080/15265161.2015.1062163. PMC4821628 26305741

[21] Chou WS, Gaysynsky A. A relationship-centered approach to addressing mistrust. J Commun Healthc. 2023;16(4):320–3. 10.1080/17538068.2023.2258683. 37732639

[22] Tamlyn AL, Tjilos M, Bosch NA, et al. At the intersection of trust and mistrust: A qualitative analysis of motivators and barriers to research participation at a safety-net hospital. Health Expect. 2023;26(3):1118–26. 10.1111/hex.13726. PMC10154811 36896842

[23] Penckofer S, Byrn M, Mumby P, Ferrans CE. Improving subject recruitment, retention, and participation in research through Peplau’s theory of interpersonal relations. Nurs Sci Q. 2011;24(2):146–51. 10.1177/0894318411399454. PMC3687539 21471039

[24] George C, Raymond KF, Collins L, Arefy Z, Kazmerski TM. Partnership Enhancement Program: Piloting a Communication Training Program for Cystic Fibrosis Care Teams. J Patient Experience. 2021;8:23743735211014049. 10.1177/23743735211014049. PMC8205322 34179443

[25] Weiss EM, Duenas DM, Kelsh A, et al. Piloting the better research interactions for every family (BRIEF) researcher intervention to support recruitment for a neonatal clinical trial: parent experience and infant enrollment. J Perinatol. 2025;45(6):773–89. 10.1038/s41372-025-02245-w. PMC12752018 40038540

[26] Weiss EM, Gray MM, Ko LK, Duenas DM, Oslin E, Kraft SA. Development of the Better Research Interactions for Every Family (BRIEF) intervention to support recruitment for neonatal clinical trials: an intervention mapping guided approach. Trials. 2024;12(1):610. 10.1186/s13063-024-08446-6. PMC11395641 39267164

[27] Kraft SA, Porter KM, Shah SK, Wilfond BS. Comprehension and Choice Under the Revised Common Rule: Improving Informed Consent by Offering Reasons Why Some Enroll in Research and Others Do Not. Am J Bioeth. 2017;17(7):53–5. 10.1080/15265161.2017.1328535. 28661747

[28] Standardizing Treatments for Pulmonary Exacerbations A Platform for Evaluating Treatment Decisions to Improve Outcomes (STOP360) Aminoglycoside Study (AG Study). National Library of Medicine (US). https://clinicaltrials.gov/study/NCT05548283.

[29] Stacey D, Légaré F, Boland L, et al. 20th Anniversary Ottawa Decision Support Framework: Part 3 Overview of Systematic Reviews and Updated Framework. Med Decis Making. 2020;40(3):379–98. 10.1177/0272989x20911870. 32428429

[30] Koyfman SA, Reddy CA, Hizlan S, Leek AC, Kodish AE. Informed consent conversations and documents: A quantitative comparison. Cancer. 2016;122(3):464–9. 10.1002/cncr.29759. PMC4724216 26505269

[31] Wall LK, Pentz RD. Another look at the informed consent process: The document and the conversation. Cancer 2016/02/01. 2016;122(3):352–4. 10.1002/cncr.29760. 26505483

[32] Sanderson SC, Lewis C, Patch C, Hill M, Bitner-Glindzicz M, Chitty LS Opening the black box of informed consent appointments for genome sequencing: a multisite observational study. Genet Med. 2019;21(5):1083–91. . 10.1038/s41436-018-0310-3. PMC6752270 30270361

[33] Hall MA, Camacho F, Lawlor JS, Depuy V, Sugarman J, Weinfurt K. Measuring trust in medical researchers. Med Care. 2006;44(11):1048–53. 10.1097/01.mlr.0000228023.37087.cb. 17063137

[34] Joffe S, Cook EF, Cleary PD, Clark JW, Weeks JC. Quality of informed consent: a new measure of understanding among research subjects. J Natl Cancer Inst. 2001;17(2):139–47. 10.1093/jnci/93.2.139. 11208884

[35] Elwyn G, Barr PJ, Grande SW, Thompson R, Walsh T, Ozanne EM. Developing CollaboRATE: a fast and frugal patient-reported measure of shared decision making in clinical encounters. Patient Educ Couns. 2013;93(1):102–7. 10.1016/j.pec.2013.05.009. 23768763

[36] Coleman C, Hudson S, Pederson B. Prioritized Health Literacy and Clear Communication Practices For Health Care Professionals. Health Lit Res Pract. 2017;1(3):e91–9. 10.3928/24748307-20170503-01. PMC6607795 31294254

[37] Hashim MJ. Patient-Centered Communication: Basic Skills. Am Fam Physician. 2017;1(1):29–34. 28075109

[38] Miller AN, Zraick R, Atmakuri S, et al. Characteristics of teach-back as practiced in a university health center, and its association with patient understanding, self-efficacy, and satisfaction. Patient Educ Couns. 2021;104(11):2700–5. 10.1016/j.pec.2021.04.041. 34400041

[39] Seely KD, Higgs JA, Nigh A. Utilizing the teach-back method to improve surgical informed consent and shared decision-making: a review. Patient Saf Surg. 2022;5(1):12. 10.1186/s13037-022-00322-z. PMC8897923 35248126

[40] Knapke JM, Hildreth L, Molano JR, et al. Andragogy in Practice: Applying a Theoretical Framework to Team Science Training in Biomedical Research. Br J Biomed Sci. 2024;81:12651. 10.3389/bjbs.2024.12651. PMC11008574 38605981

[41] Wijnen-Meijer M, Brandhuber T, Schneider A, Berberat PO. Implementing Kolb´s Experiential Learning Cycle by Linking Real Experience, Case-Based Discussion and Simulation. J Med Educ Curric Dev. 2022;9:23821205221091511. 10.1177/23821205221091511. PMC9112303 35592131

[42] Li LC, Grimshaw JM, Nielsen C, Judd M, Coyte PC, Graham ID Evolution of Wenger’s concept of community of practice. Implement Sci. 2009;4(1):11. . 10.1186/1748-5908-4-11. PMC2654669 19250556

[43] Morain SR, Barlevy D, Joffe S, Largent EA, Physician-Investigator. Research Coordinator, and Patient Perspectives on Dual-Role Consent in Oncology: A Qualitative Study. JAMA Netw Open. 2023;3(7):e2325477. 10.1001/jamanetworkopen.2023.25477. PMC10369198 37490290

[44] Morain SR, Joffe S, Largent EA. When Is It Ethical for Physician-Investigators to Seek Consent From Their Own Patients? Am J Bioeth. 2019;19(4):11–8. 10.1080/15265161.2019.1572811. 30994425

[45] Childress JF. The Place of Autonomy in Bioethics. Hastings Center Rep. 1990;20(1):12–7. 10.2307/3562967. 2179164

[46] Horng S, Grady C. Misunderstanding in clinical research: distinguishing therapeutic misconception, therapeutic misestimation, and therapeutic optimism. IRB Jan-Feb. 2003;25(1):11–6. 12833900

[47] Kim SY, Miller FG. Informed consent for pragmatic trials–the integrated consent model. N Engl J Med. 2014;20(8):769–72. 10.1056/NEJMhle1312508. 24552326

[48] Glanton E, Yarrington CD, Calderwood L, Torrey Sosa L, Connors PD. The importance of personalismo: Navigating obstacles in recruitment strategies for Spanish speakers in marginalized communities. J Genet Couns. 2020;29(3):391–8. 10.1002/jgc4.1240. 32144873

