# Supplementary material for: Supporting high-quality enrollment decision-making in clinical research: development of a novel informed consent communication framework and curriculum
Source: BMC Med Res Methodol. 2026 Jul 25;26:231. doi: 10.1186/s12874-026-02885-2 (PMC13721633; doi:10.1186/s12874-026-02885-2)
Supplement: Supplementary file 1 [file 12874_2026_2885_MOESM1_ESM.pdf]

THE RIVER APPROACH

WHAT IS IT? A relationship-based approach to informed consent communication that is designed to support values-aligned enrollment decisions.

- **R** RELATIONSHIP
- **I** INFORMATION
- **V E** VALUES EXPLORATION
- **R** RESOLUTION

HOW TO USE THIS GUIDE

- This guide offers example questions for each step in RIVER. **It can be used with any consent form.**
- **These questions should be tailored for each potential participant and the specific study context.** It is important to go through each step, but you may add, skip, or modify questions as needed. You may also need to revisit steps.
- People will have different preferences for how in-depth a discussion they want to have. Be sure to give everyone the opportunity to learn about the study and explore their values. **Use your judgment to adjust your approach based on each person's preferences.**

ABOUT THE ENVISION PROJECT

The ENVISION project (Engaging with Values to Inform research enrollment deciSIONs) aims to improve values-aligned decisions about research enrollment.

We are doing this by developing informed consent tools, like this discussion guide, to support research teams as they approach potential participants. We partner with clinical trials to accomplish these goals.

ENVISION is funded by the Cystic Fibrosis Foundation.

For more information about ENVISION, go to envisionconsent.org/stop360 or scan the QR code below.

ENVISION
engaging with values to inform research enrollment decisions

INFORMED CONSENT DISCUSSION GUIDE

The RIVER Approach

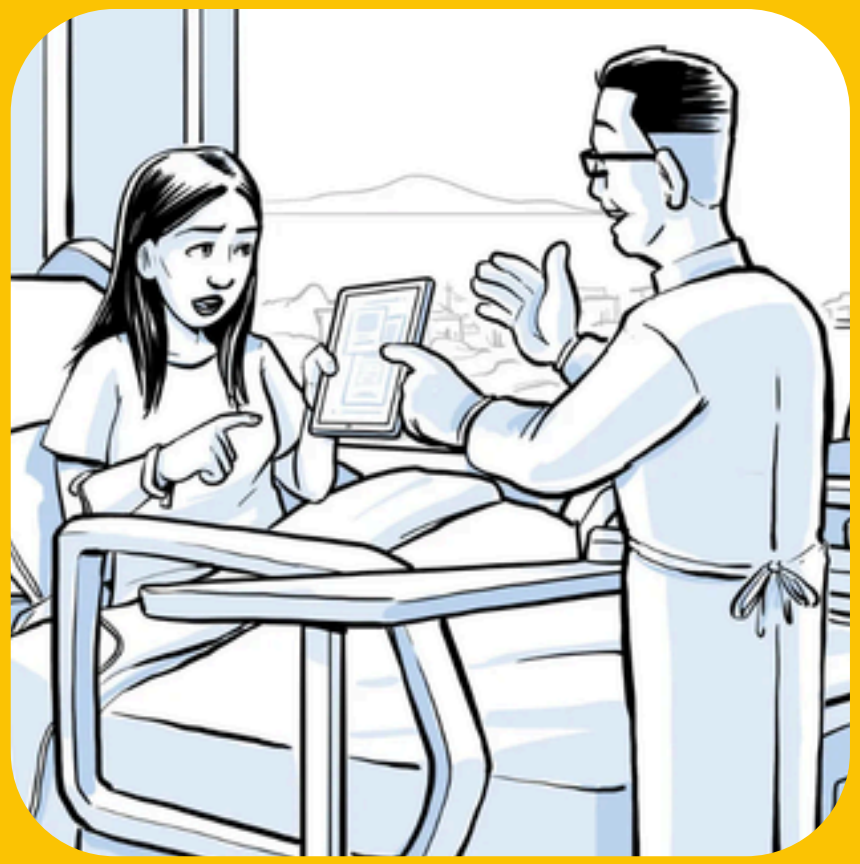

© 2025 by Booster Shot Media, Inc.

THE RIVER APPROACH

RELATIONSHIP | **I**NFORMATION

VALUES **E**XPLORATION | **R**ESOLUTION

RELATIONSHIP

Introduce yourself to everyone in the room. Avoid making assumptions about people's relationships.

- Hi, I'm [name and role]. I'm here to talk with you about a potential research study.
- Are you [name]? It's nice to meet you. How would you like me to address you?
- Who have you brought with you?

Acknowledge the potential participant's **personal and clinical situation** and share that you are there to support them.

- I understand from your doctor that [clinical status]. How are you feeling about this? What previous experiences have you had with this?
- Your doctor, [name], shared that there is a research study that you might be interested in. I'm here to talk with you about the study and support you in thinking about whether it might be a good fit for you.

Check if it's a **good time to talk** about research. If it's not, set a time to follow up.

- Would you be interested in talking with me about this research study?
- This discussion usually takes about [estimated time]. Is this a good time?
- What other things do you want to attend to before we talk?

INFORMATION

Review the informed consent in **small sections**. Explore topics the potential participant is most interested in and check in on their **understanding**.

- I'm curious to hear what stood out to you about the information we've reviewed so far.
- To help me be sure I'm explaining everything as clearly as I intend, would you mind sharing in your own words what we've covered so far?

Elicit and list the potential participant's **questions**. Spend more time on topics where they have questions and check in after key parts.

- I want to take some time to check what questions you have. People have questions about lots of different things in the study, such as how much time it takes or the risks involved. Let's make a list of your questions so we can make sure we address all of them.

VALUES EXPLORATION

Explore the potential participant's values that relate to whether they might want to join or not. Support them in reflecting on what they share and thinking through how that might shape their decision.

As you review the study information, explore what **reasons** they are thinking about joining or not.

- We discussed a few examples of reasons why people might want to join or not join the study. Which reasons resonate with you?
- It sounds like [reflect reasons] are important to you. Do these make you lean in one direction or the other at this point?

Elicit **priorities** that might be relevant to the study. These often include health-related priorities and other life priorities during the study period.

- I'm interested in hearing what you're thinking and how this study might/might not fit into your life right now. When you think about whether this study is something you want to take part in, what things are important to you?
- Thanks for sharing [reflect]. If you imagined joining the study, how would that fit with what you've shared with me?

Elicit any **concerns or barriers** to participation and discuss any ways these could be navigated.

- How would the schedule of this study work for you? What could we do to help with this?
- What is your support system for participating in this study? How will you get to visits? What could we do to help with this?
- Is there anything else you've heard so far that might be an issue for you?

RESOLUTION

Reflect, confirm, and validate the person's key reasons, priorities, and concerns/barriers (and ways to address them). Check in on their decision.

- We've talked about [reflect]. Did I get that right? Do you want to talk more about anything?
- How are you feeling about the study?

If they ...

SAY YES	**Thank them** for the conversation and **provide them next steps** for the study.
SAY NO	**Thank them** for their time/consideration.
EXPRESS CONFLICT	**Validate** their uncertainty and **further explore** their values.
NEED TIME	**Check in** on any next steps they may want to take before deciding.
